# Commentary: From education to exploitation: the high price paid by resident physicians in Ecuador's medical specialization

**DOI:** 10.3389/fmed.2025.1550144

**Published:** 2025-02-06

**Authors:** Iván Cevallos-Miranda, Gonzalo Mantilla, Ivan Sisa

**Affiliations:** ^1^Universidad San Francisco de Quito USFQ, Colegio de Ciencias de la Salud, Escuela de Especialidades Médicas, Quito, Ecuador; ^2^Universidad San Francisco de Quito USFQ, Colegio de Ciencias de la Salud, Escuela de Medicina, Quito, Ecuador

**Keywords:** medical training, postgraduate medical education, health policy, health care workforce, Ecuador

## Introduction

We read with interest the opinion article by Izquierdo-Condoy et al. (1) regarding the significant shortage of specialist physicians in Ecuador and how this scenario has evolved to a form of systemic exploitation with overworked and poorly economically compensated. Currently, in Ecuador, there are 62 universities accredited by our local Higher Education Council (CES) (34 public and 28 private universities) (2). However, only 16 universities have programs for medical specialization, and 10 are private universities. Jointly, these universities offer 36 residency programs or postgraduate training where Ecuadorian physicians can obtain a medical specialization (Table 1).

**Table 1:** Accredited Ecuadorian medical residency programs by higher education institutions (public and private).

**Residency program**	**PUCE**	**UCACUE**	**UCE**	**UCSG**	**UCUENCA**	**UDA**	**UDLA**	**UEES**	**UG**	**UIDE**	**ULEAM**	**UNL**	**USFQ**	**UTA**	**UTE**	**UTPL**
Anesthesiology	5	–	–	1	1	–	1	1	1	–	–	–	1	–	–	–
Cardiology	1	–	–	–	–	–	1	1	–	–	–	–	–	–	–	–
Plastic surgery	1	–	–	–	–	–	–	–	–	–	–	–	–	–	–	–
Palliative care	1	–	–	1	–	–	–	–	–	–	–	–	–	–	1	–
Coloproctology	1	–	–	–	–	–	–	–	–	–	–	–	–	–	–	–
Geriatrics and gerontology	5	–	–	–	–	–	–	–	–	–	–	–	–	–	–	–
Infectology	1	–	–	–	–	–	–	–	–	–	–	–	–	–	–	–
Emergency medicine	3	–	–	–	1	1	–	1	–	–	–	–	–	–	–	–
Internal medicine	1	–	–	–	1	–	–	1	–	1	–	–	–	1	–	–
Pediatrics	6	1	–	1	1	1	1	–	–	1	–	–	–	–	–	–
Angiology and vascular surgery	1	–	–	–	–	–	–	–	–	–	–	–	–	–	–	–
General surgery	1	–	1	–	1	–	1	1	–	1	–	–	–	–	–	–
Orthopedics and traumatology	1	–	1	–	1	–	1	1	–	1	–	–	1	–	–	–
Gastroenterology	1	–	–	–	1	–	–	–	–	–	–	–	–	–	–	–
OB/GYN	1	1	1	1	1	1	1	–	–	1	–	–	–	–	–	–
Critical care medicine	5	–	1	–	–	–	1	1	–	–	–	–	–	–	–	–
Family medicine	5	–	1	1	–	1	–	–	–	–	–	1	–	–	–	1
Otolaryngology	1	–	–	–	–	–	–	1	–	–	–	–	–	–	–	–
Oral and maxillofacial surgery	–	–	1	–	–	–	–	–	–	–	–	–	1	–	1	–
Dermatology	–	–	1	1	–	–	–	1	–	–	–	–	–	–	1	–
Imaging	–	–	1	–	1	–	–	1	–	1	–	–	1	–	–	–
Nephrology	–	–	1	–	–	–	–	1	–	–	1	–	–	–	–	–
Pneumology	–	–	1	–	–	–	1	–	–	–	–	–	–	–	–	–
Ophthalmology	–	–	1	1	–	–	–	–	–	–	–	–	–	–	1	–
Psychiatry	–	–	1	–	1	–	–	–	–	–	–	–	–	–	1	–
Radiation oncology	–	–	1	–	–	–	–	–	–	–	–	–	–	–	–	–
Pediatric surgery	–	–	–	1	–	–	–	–	–	–	–	–	1	–	–	–
Pediatric critical care medicine	–	–	–	1	–	–	–	–	–	–	–	–	1	–	–	–
Neonatology	–	–	–	1	1	–	–	–	–	–	–	–	–	–	1	–
Rheumatology	–	–	–	–	1	–	–	–	–	–	–	–	–	–	–	–
Endocrinology	–	–	–	–	–	–	1	–	–	–	–	–	–	–	–	–
Occupational medicine	–	–	–	–	–	–	1	–	–	–	1	–	–	–	–	–
Neurology	–	–	–	–	–	–	1	–	–	–	–	–	–	–	–	–
Urology	–	–	–	–	–	–	–	1	–	–	–	–	–	–	–	–
Neurosurgery	–	–	–	–	–	–	–	–	–	–	–	–	1	–	–	–
Rehabilitation medicine	–	–	–	–	–	–	–	–	–	–	–	–	–	–	1	–
**Total**	41	2	13	10	12	4	11	12	1	6	2	1	7	1	6	1

This table was built with information from the Ecuadorian Higher Education Council (https://appcmi.ces.gob.ec/oferta_vigente/index.php) and shows the number and specialty of the residency programs available per university in Ecuador.

OB/GYN, Gynecology and Obstetrics; PUCE, Pontifical Catholic University of Ecuador; UCACUE, Catholic University of Cuenca; UCE, Central University of Ecuador; UCSG, Catholic University of Santiago de Guayaquil; UCUENCA, University of Cuenca; UDA, University of Azuay; UDLA, University of the Americas; UEES, Holy Spirit University of Specialties; UG, University of Guayaquil; UIDE, International University of Ecuador; ULEAM, “Eloy Alfaro” Secular University of Manabí; UNL, National University of Loja; USFQ, San Francisco University of Quito; UTA, Technical University of Ambato; UTE, UTE University; UTPL, Private Technical University of Loja.

## Discussion

It is well-known the scarcity of specialist doctors especially in low- and middle-income countries (LMICs) and the difficulties they must face to get a medical specialization. For example, LMICs represent 48% of the global population but have 20% of the surgical specialist workforce compared to high-income countries (3). The present article focuses on this systemic and global health problem taking Ecuador as a case sample based on three aspects: (i) the status of physicians in training as “students in training” which impedes them from receiving a regular employee salary within the Ecuadorian public health sector, (ii) the scarcity of residency programs outside major cities, and (iii) physical and mental health risks due to excessive workweeks that go beyond 100 h. This could ultimately have negative impact on the medical system and healthcare in general. For example, a study found that intensive care unit residents made more serious medical errors under extended shift schedules than with reduced work hours per week (4). Furthermore, Izquierdo-Condoy et al. advice that incorporating perspectives from local key stakeholders in charge of the training of young Ecuadorian medical graduates could enhance the depth/impact of this analysis (1). Based on our own experience training resident physicians at San Francisco University of Quito (USFQ, Spanish acronym), we would like to extend this extremely important discussion. In the Ecuadorian case, the most outstanding point revolves around remuneration. Hence, meeting economic needs is without a doubt a priority for resident physicians because it is the pivot for their mental health and performance in their residency program. However, the context is much broader and necessarily involves understanding the economic reality of Ecuador, which is very different from that of other countries such as the United States, the United Kingdom, or Australia, with an average gross domestic product per capita of US$57,198 compared to US$6,455 of Ecuador (5). Despite of this even in high-income countries, cases of medical exploitation have been reported among physicians in training. For example, last December ~400 residents and fellows in training in Boston, Massachusetts protested outside one of the world-renowned hospitals in the area. The protestants claimed that they do not receive fair economic compensation for the long week hours they work (6). Higher levels of stress, anxiety, and depression measurements, related to workload, have been found in medical specialties including anesthesiology, general surgery, gynecology, and intensive care medicine, in which due to the nature of the clinical practice, patients' expectations, and the critical decisions to be made put at higher risk to burnout compared to other specialties, such as dermatology, psychiatry, family medicine, or pathology (7, 8). Thus, the quality of care is impacted by the above, which is added to the lack of specialists at a time when medicine requires the highest specialization to benefit patients. However, the literature informs an excessive training of general practitioners, overcrowding in some specialties and serious deficiencies in others (9). How can all the interests involved in this problem be harmonized? That is the question we must answer between stakeholders involved including government, health institutions, and universities. The USFQ has participated in concrete proposals to achieve regulatory changes and has worked positively with the Ecuadorian Ministry of Public Health. Thus, we propose some initiatives to bring potential solutions to tackle this public health issue that not only affects Ecuador but also most LMICs:

1. Create a mental health profile at entry to the medical and postgraduate programs and periodically monitor them during their advancement and graduation. In addition, make available physician wellness programs with 24/7 access to psychologists skilled in physician mental health (10).
2. Define locally a fixed number of graduates annually in medicine, otherwise could be a potential source of labor exploitation.
3. Differentiate between specialization programs to establish the workloads in the hospital facilities.
4. Analyze the duration of the different programs so that the workload, the teaching activity, and compliance with the number of procedures according to the profile of the specialist's graduate can be balanced.
5. It should be considered that specialties that require practical skills, such as surgery, if hospital practice hours are reduced, will have to extend the duration of the program or reduce the number of students. This would lead to increases in the costs of education and in the useful life of the specialist.
6. Guide local specialty requirements based on a well-planned national policy addressing issues of access and equity (9).
7. Support the Ecuadorian Ministry of Public Health initiatives to change the current legal status of students in training to physicians in training so they are able to receive a regular employee salary in the public sector (11).
